# Lecture on Clinical Medicine, Delivered at the Philadelphia Medical Institute

**Published:** 1838-09-26

**Authors:** W. W. Gerhard

**Affiliations:** Physician to the Philadelphia Hospital, &c.


### CLINICAL LECTURE.

### LECTURE ON CLINICAL MEDICINE, *delivered at the Philadelphia Medical Institute, by* W. W. Gerhard, M. D., *Physician to the Philadelphia Hospital*, &c.

### ACUTE INFLAMMATIONS OF THE MEMBRANES OF THE HEART.

During the past summer I have very frequently alluded to the inflammations of the membranes of the heart. It has so happened that we have had a very unusual number of these diseases; you have seen more cases of the kind within the last six months than I have observed within the last two or three years, and it is very improbable that you will again witness so many cases of these affections, in the same short space of time. The serous inflammations have been almost endemic in our city, and, if we except a moderate proportion of bowel affections, they have constituted the prevailing diseases of this period.

It was for this reason that I commenced the course with acute rheumatism, and then passed on to the consideration of the serous inflammations so closely connected with it. Amongst these, the subject of the membranous inflammations of the heart was accidentally introduced; but I did not then point out to you the numerous interesting questions arising from the study of these inflammations, which you will find of difficult diagnosis, unless you are thoroughly acquainted with their pathology and with the means of physical exploration. Without the aid of the physical signs of disease, the inflammations of the heart cannot be recognised, except in a very small proportion of cases. You have had a recent illustration of this fact: a patient, whose case I shall presently detail you, has been evidently labouring under pericarditis of considerable intensity; the effusion of lymph and serum occurred, as it were, under our own eyes, and you were able to trace the gradual consolidation of the lymph, when adhesions began to form between the two surfaces of the pericardium, yet the patient has complained of no pain whatever in the chest, and no uneasiness other than that caused by the rheumatic inflammation of the joints which preceded the pericarditis. In but two or three cases was the pain sufficiently considerable to induce us to suspect the occurrence of any affection of the heart; one of these cases, attended with pains and the best marked, was that of the man Robb, which I mentioned when speaking of inflammatory rheumatism. He suffered some pain, but usually inconsiderable, until his entire recovery. The other two were blacks, who were affected with inflammation of both membranes of the heart, and recovered, but died afterwards of a consecutive dropsy. These latter patients had recovered entirely of the cardiac affection, and, in all probability, would not have fallen victims to the dropsy had they not both laboured under a cancerous disease.

When you examine more fully the history of the cases, you will find the other rational signs equally obscure; we can affirm that the obscurity was not owing to our want of appreciation of these signs, for I examined the cases in your presence with the most scrupulous accuracy, and you can bear witness to the precautions taken to elicit every practicable symptom in the case. The inflammations of the heart are, therefore, to a great degree, latent, and you must commence their study, with the conviction that their diagnosis is impossible, in a large proportion of cases, without a thorough knowledge of their pathology and a sufficient acquaintance with the physical signs of disease. But if this knowledge be possessed, and some of you have already attained it, there is no part of medicine more perfectly demonstrative in its character, or which is governed by more unvarying laws, as to its progress and termination.

I will now relate to you some of these cases and we can compare them together to ascertain how these remarks are confirmed or invalidated by your own observation. At all events you have been eye-witnesses of what I shall narrate to you, and the circumstances of the cases will, therefore, be more completely impressed upon your mind, and will carry with them a force of conviction and a clearness of detail, which could never result from a purely didactic lecture.

The first case I shall give you is that of a patient, now convalescing in our wards from pericarditis, nearly uncomplicated with inflammation of the internal membrane of the heart. It occurred, as is most commonly the case, during an attack of acute inflammatory rheumatism, and had just appeared when the patient came under your observation. The following symptoms were dictated in your presence:—

"David Dargan, aged thirty-eight, a lime-burner, accustomed to drink freely, entered my ward September the 2d. On the 28th of August, after drinking rather more freely than usual, he was taken with convulsions and became slightly deranged; he was bled, returned for a short time to consciousness, and again became incapable of recollection. On the 29th he was stupid and could answer no questions; the stupor was unaccompanied by distortion or active delirium. No muttering; no return of convulsions. Digestive functions good. He was cupped freely to the nucha twice; pediluvia were applied; he took nitre and the effervescing mixture, and was purged. In two days he recovered his intellect, but not entirely his memory. Consciousness not quite perfect until the 2d. On his return to consciousness, he had pains in both legs, hands, and shoulders, with swelling. From the 1st to the 2d there was increase of pain and heat, and on the 2d there was redness. No edema nor palpitations. There was intense cephalalgia, which was relieved by cupping. For the last three or four years he had fluttering of the heart after exercise, and was short-breathed at the same time. This began after an illness of seven months duration, of intermittent fever. He had rheumatism ten years ago, after a wetting, and two or three times before, but merely local in the shoulders, not confining him to bed. He recollected no other illness, never had syphilis. At the beginning of the pains, on the 28th, had a severe chill, but none after.

"His condition, on the 2d, was as follows; swelling, heat, and pain in the ankles and feet; slight swelling of the knees; and redness and swelling of the hands in nearly all the joints. Some pain, but no swelling, in both shoulders, but none in the elbows. Pulse ninety, full, nearly regular. Tongue moist and natural. Appetite good; no nausea; stools regular. Skin generally warm, scarcely moist. Both sounds of the heart heard in the whole præcordial region, varying, but never perfectly natural. The first sound more or less roughened, the second heard distinctly, rather sharp; between the two, or rather, at the commencement of the second, was heard a very evident bruit de frottement, which could be detected over the whole præcordial region, but was more distinct over the left margin of the sternum, more marked in the erect than in the recumbent posture. Impulse of the heart increased and irregular. Percussion dull from the right margin of the sternum to the nipple, and from the fourth rib downwards. No pain, no dyspnœa, almost no cough, no uneasiness on percussion. Spinal tenderness from the seventh dorsal vertebra to the fifth cervical, more severe on the spine than on the adjoining parts. Twenty cups were ordered to the spine, with a sixth of a grain of tartar emetic and opium each, every two hours, and low diet.

"On the 3d, there was great alleviation of the rheumatic symptoms. No cough or pain across the chest; no palpitation. Creaking sound in the præcordial region more distinct than on the 2d, extending over the whole region, and synchronous with the diastole, varying in intensity and tone. Impulse of the heart diminished, more diffused. Sound much less loud, and both heard distinctly, the first less rough than on the 2d. Prominence rather greater. Percussion dull to an inch and a half beyond the nipple, thence to right margin of the sternum. Respiration posteriorly vesicular throughout the chest, resonance of the voice doubtful; opium and tartar emetic continued, twice the quantity of the former, with cups to the præcordia.

"On the 4th, the pain and swelling were rather less, but there was great weakness, which may have been owing to the tartar emetic. Other symptoms better. Eight ounces of blood had been taken from the chest, by the cups. Percussion now quite clear within the nipple, dulness extending merely an inch and a quarter from mid-sternum. Sounds of the heart much louder, the first offering only a moderate bruit de soufflet; the second, near the point of the sternum almost converted into a simple creaking sound, which extended also to the first, but less distinctly; heard all over the heart, less towards its left margin, very loud along its whole sternal region, at times giving a musical tone. When the patient was erect, the impulse of the heart was stronger, creaking more frequent, converted into an incessant grating. Eight ounces of blood were again taken from the præcordia by cups, and the opium and tartar emetic continued.

"On the 5th, the pain was less severe in the hands, more so in the shoulders and muscular parts of the arms; soreness in the muscles of the thighs, less in the feet; increase of swelling and puffiness in the knees, but not of pain and swelling. No spinal tenderness, cough, or oppression; sleep disturbed by pain; prominence greater than yesterday; percussion clear, however, except for an inch and a half at the point of the sternum; impulse of the heart greater, clearer, sharper; first sound diminished, grating much less distinct, heard chiefly at the point of the sternum. On sitting up, the action of the heart was stronger, the grating much more distinct, heard under the same circumstances as before; three stools since last evening; last evening took a half a grain of ipecacuanha and five grains of Dover's powder, twice. To-day, five grains of Dover's powder, four times a day, and cups to the spine.

"On the 6th, no swelling in the knees, almost no soreness, less of both in the feet, none in the left hand, almost none in the right; soreness and swelling in the shoulders not diminished; no soreness of the back or breast. Pulse ninety-two, regular, and softer. Sweating profuse, no chilliness. First sound of the heart very short and faint. Second, loud, marked by the creaking sound. Percussion duller, to an inch within the nipple; impulse rather stronger. Cups between the shoulders, and Dover's powder continued.

"On the 7th, countenance gay; shoulders better; no swelling of the hands, almost no stiffness; knees natural; very slight swelling in the ankles, with a little pain in the right. No spinal tenderness. (Has been cupped four times, twice to præcordia, eight ounces, each time, twice to spine, six ounces, twice cupped in the cells to the nucha, seven ounces, and had been bled before he came in from arm.) Pulse quick, regular, ninety-five. No prominence in the præcordia. Percussion perfectly clear. Dulness very incomplete every where. Impulse of the heart more clear; first sound prolonged; creaking in the second limited to the point of the sternum, disappearing when he rises. Sweating profuse, constant. Urine rather increased. No chills. Dover's powder continued; hop poultice to the feet.

"On the 8th, soreness almost ceased in the feet, a little effusion in the knees, but no increase of pain; slight soreness at the points of both shoulders. Pulse one hundred and eight, thrilling, regular. Sweating continues. Impulse of the heart much stronger, first sound nearly natural, a little prolonged; cri du cuir so faint at the beginning of the second as to be doubtful, if not previously heard. Treatment continued, with cups between the shoulders.

"On the 9th, has pain only in the knees, and hips; less in the shoulders since the cupping. Sweating still continues. Pulse one hundred and two, quick, thrilling, and resisting. Appetite good; no nausea; three or four stools daily. Respiration now heard over the whole præcordial region; impulse stronger, creaking quite decided in the second sound; first still blowing, less than last evening, when the pulse rose ten to fifteen beats. Dry cups were applied to knees last evening with relief to the pain. Treatment continued.

"On the 11th, no pain in the hands; some cephalalgia. Pupils a little contracted. Some wrinkling of forehead. Expression anxious. Pulse one hundred, full, thrilling, softer than yesterday. Slight subsultus; talking in sleep, says he is accustomed to it when well; sweating continues; five stools in the twenty-four hours; legs restored to motion, almost no swelling; stiffness of right shoulder and arm, including elbow; slight of left; feels no uneasiness in the chest; a little soreness, apparently in the pectoral muscle of both sides; first sound of the heart and impulse natural; creaking scarcely heard; (has taken no medicine in the last twenty-four hours,) four ounces of wine in whey, and an assafœtida plaster to the epigastrium.

"12th.—Last evening, about seven o'clock, had more tremor, more subsultus, countenance the same; took four ounces of the assafœtida mixture, every two hours. Enema of twenty drops of laudanum. Slept well during the night, awoke once or twice only. Pulse ninety-six, full, soft; pains not increased. Soreness felt now only in the shoulder joints. Bruit de soufflet harsher than yesterday; a little rasping, creaking indistinct; second sound very clear. Percussion nearly as before, a little less clear; assafœtida mixture continued; wine omitted; full diet.

"13th.—Muttering during sleep; pain less; two stools in twenty-four hours. Impulse of the heart louder and clearer; both sounds louder, particularly second, which is still a little bellows, still subsultus; sweating. Pulse ninety-two, feeble, regular; continue assafœtida.

"17th.—Still has pain in legs and arms; drowsiness constant; no subsultus; intellect quite clear. Pulse one hundred, regular, small; motion returned to every joint, but some stiffness in shoulders and knees; sweating abundant. Both sounds of heart heard; creaking not ceased; more diffuse, less loud. Percussion not increased. Chamomile tea.

"18th.—Sitting up; no pain except slight in shoulders and knees; sounds of heart natural, except slight creaking in second. Convalescence confirmed.

"19th.—Perfectly free from pain, except when moving; then suffers from soreness of limbs; skin cool, pleasant; appetite good; sounds of the heart clear; creaking barely susceptible.

"20th.—Continues well; remains a few days longer to confirm his strength."

When you examine the history and progress of this case, you will see upon what facts the diagnosis of pericarditis is based. We must then examine other cases which have terminated fatally, in order to test the correctness of the laws of diagnosis, which I shall lay down. We have, fortunately, lost no case in the present course, during the existence of the pericarditis; but we shall be able to obtain the necessary evidence from an examination of those cases which terminated fatally of some accidental disease after the termination of the pericarditis, and we then can compare those cases with others that have terminated unfavourably at a previous period.

The signs of pericarditis in this case varied according to the stage of the disease. During the period of effusion, which had already begun when the patient came under our observation, five or six days after the commencement of the rheumatism, we had the physical signs of pericarditis, which are clearly described by Dr. Louis. That is, flatness on percussion to a much greater extent and to a more considerable degree than occurs in a healthy subject, decided prominence of the præcordial region which was distended and raised up by the liquid, and dulness of the sounds of the heart, and feebleness of impulse. Now, these signs become the more characteristic, from their frequent variation; the quantity of liquid scarcely remained the same for two consecutive days, and you, therefore, found the signs of the disease to increase during the time that it advanced, while they diminished very rapidly when the pericarditis declined. Had the dulness and the prominence of the præcordial region been permanent, the cure would still have been evident, but there might have remained some room for doubt; for chronic enlargement of the heart, particularly if complicated with effusions of serum into the pericardium, will resemble somewhat a case of pericarditis. The resemblance ceases when you watch the case for several consecutive days.

There was another sign indicative of pericarditis, which also served to point out to you the variety and stage of the disease. It was the sound produced by the rubbing together of the two surfaces of the pericardium covered with lymph. This sound occurs during the systole and diastole of the heart, especially the latter, it was, therefore, most evident in the second sound of the heart which occurs during its dilation. This new sound was so loud, as, in some measure, to conceal the natural second sound of the heart, which was, however, never entirely destroyed; it could always be detected by a practised ear, as it were, combined with the new adventitious sound. The second cardiac sound was not lost, because it depends on valvular contraction, and the valves of the heart remained nearly in the normal state; now, had the disease been complicated with much inflammation of the internal membrane, as was the case with the man Robb, to which I have already alluded, the motion of the valves would no longer have remained free, and we should have found either that the second sound was altogether lost, or much changed from its natural character. The cause of the grating of sound is nearly the same in inflammation of the pericardium and of the pleuræ; that is, in both cases it arises from the friction of two surfaces of serous membranes more or less coated with lymph; it varies somewhat, because the quick action of the heart differs greatly from the slow gradual movement of the lungs in the act of respiration. The grating of sound of pericarditis, therefore, becomes more sharp and quick, but less loud and prolonged than that of pleurisy. It is useless to describe this sound to you, for you have heard it for yourselves, which answers better than any description; those who have not heard it, may readily distinguish it by its creaking, like the sound produced by rubbing together two pieces of moist leather, whence it has been sometimes called the "bruit de cuir," or leather sound; a trivial name, which is by no means so expressive, as that of rubbing or grating sound. It cannot be recognised by one not previously acquainted with the natural sounds of the heart, with which the slighter shades of this adventitious sound may be confounded. You could distinguish it readily in the present instance by a careful analysis of the sounds, when you found that the sharp clear tone of the second sound was more or less concealed by this rasping sound extending over the whole anterior surface of the heart, especially at its thickest portion. There is but little difficulty in distinguishing the grating sound of pericarditis from the rasping sound caused by thickening or vegetations upon the valves; the latter sound is most frequent in the systole, is always heard most distinctly at the region of the valves, and is not attended with a sensation of glowing, which is quite perceptible to the touch in most cases of pericarditis.

As the lymph became consolidated, the grating sound gradually declined, but it has not yet disappeared, although the patient is now in full convalescence. Nor do you generally find that this sound will disappear quickly; for, if the lymph form prominences on the surface of the heart, it may persist for several months at least, until they are so far absorbed as no longer to present rough projections for the grating of the two surfaces together.

The sounds of the heart, properly so called, were both distinguishable throughout the whole of this case; they were somewhat feeble, had lost a little of their natural clearness and seemed distant, but neither of them was either very much changed, or had lost its due proportion. In simple pericarditis, you will find that this is usually the case. It is true, that the motion of the heart is never so free as to offer its sounds in their full degree of developement, but it is also true, that the slight aberration from the normal sound which does occur in simple pericarditis, is very different from the rasping or very rough bellows sound heard in cases of endocarditis; whether this latter disease be simple or merely a complication of the pericarditis. You had a beautiful illustration of this distinction when you examined the case of the patient Robb, which was described when speaking of acute articular rheumatism. In Robb there was both endo and pericarditis, and we had the distention and dulness of sound indicative of pericarditis, with the rough and harsh sound, caused by the thickening and consequent stiffness of the valves. The chain of proof of what I have just advanced is, with me, conclusive, for I have seen cases of both endocarditis and pericarditis quite uncomplicated one with the other, and, therefore, well suited for studying the signs of these diseases in their simple state. I have again seen other cases in which the symptoms of one or other disease greatly predominated, without being perfectly unmixed one with the other. Of course I am now alluding to cases which terminated fatally, and in which examination after death proved the correctness of the diagnosis. The most simple cases of heart disease of the kind to which I am now alluding were furnished by two patients, affected with pneumonia; both died of the pneumonia, which was aggravated by the disease of the heart, although this latter affection was not, in itself, sufficient to cause death. By this comparison of the symptoms, in these cases, you can retain no reasonable doubt as to the accuracy of our means of diagnosis, and the necessity of an acquaintance with pathological anatomy.

The first case occurred in the last winter; it was laryngitis, attended with extreme prostration; the patient was then attacked with pneumonia, of which he died. During the course of the pneumonia, the patient was taken with pericarditis; there was a manifest dulness in the præcordial region, a slight prominence, and a distinct, though feeble creaking sound, chiefly heard during the dilatation of the heart. The sounds of the heart scarcely differed from the normal standard, although the impulse was somewhat feeble. As there was some obscurity in the case, the patient was, at my request, examined by my colleague, Dr. Pennock, who concurred with me in the diagnosis. The patient died, some days afterwards, from the pneumonia; on the autopsy we found patches of false membrane scattered over the surface of the pericardium, proving the existence of pericarditis. There was no liquid in the pericardium; this again corresponded with the accuracy of the diagnosis, for, previously to the death of the patient, the pericarditis had evidently declined, and the dulness on percussion had been gradually replaced by the natural resonance. This case, which occurred during the course of the present winter, afforded conclusive evidence of the actual relation between the signs of pericarditis and the corresponding anatomical lesions.

The second case presented itself more recently; it was that of a man ill with pneumonia which had advanced to the period of suppuration, previously to his admission. This patient offered, during life, the signs of uncomplicated endocarditis; there was a dull, confused action of the heart, neither of the natural sounds being very distinct; their rhythm was also somewhat changed. The impulse was diffused and labouring. There was but a very slight increase of the natural dulness on percussion, at the præcordial region, and there was no creaking sound. I considered this case as one of endocarditis, without much valvular alteration, and mentioned my reasons for this diagnosis to several of you, who were then present. The patient died of the mingled effects of the pneumonia and the endocarditis, and, as you remember, we found the internal membrane of the heart reddened; and as well as that of the origin of the aorta, it was covered with a delicate membrane that could be detached from it in strips of considerable length. On examination of this membrane by the aid of a magnifying lens, we found that it was evidently organized and traversed by numerous blood-vessels.

These cases afford you the evidence which you have a right to require, and prove to you the actual connection existing between certain physical signs and the acute inflammations of the membranes of the heart. It remains for me to examine the signs of other cardiac diseases, more or less similar to those I have just described to you. It will, therefore, be necessary for me to analyse their functional, as well as physical signs; a task which I reserve for a future lecture.

---

